# Hard Tempered Drill

**Published:** 1883-10

**Authors:** 


### HARD TEMPERED DRILL.

A drill that will drill even average tempered steel can be made by heating the steel to a cherry-red and then cooling in quicksilver. Such drills are exceedingly useful in dental operations.—*Ibid.*

